# Automatic and Selective Single Cell Manipulation in a Pressure-Driven Microfluidic Lab-On-Chip Device

**DOI:** 10.3390/mi8060172

**Published:** 2017-06-01

**Authors:** Yigang Shen, Zhenyu Song, Yimo Yan, Yongxin Song, Xinxiang Pan, Qi Wang

**Affiliations:** 1 Department of Marine Engineering, Dalian Maritime University, Dalian 116026, China; dmushenyigang@163.com (Y.S.); Leonhard.Yan@outlook.com (Y.Y.); panxx@dlmu.edu.cn (X.P.); 2 Department of Radiotherapy, Jiaozhao Central Hospital, Qingdao 266300, China; qdsongzhengyu@163.com; 3 Department of Respiratory Medicine, The Second Hospital Affiliated to Dalian Medical University, Dalian 116027, China

**Keywords:** single cell manipulation, microfluidic chip, resistive pulse sensing, hydrodynamic isolation

## Abstract

A microfluidic lab-on-chip device was developed to automatically and selectively manipulate target cells at the single cell level. The device is composed of a microfluidic chip, mini solenoid valves with negative-pressurized soft tubes, and a LabView^®^-based data acquisition device. Once a target cell passes the resistive pulse sensing gate of the microfluidic chip, the solenoid valves are automatically actuated and open the negative-pressurized tubes placed at the ends of the collecting channels. As a result, the cell is transported to that collecting well. Numerical simulation shows that a 0.14 mm^3^ volume change of the soft tube can result in a 1.58 mm/s moving velocity of the sample solution. Experiments with single polystyrene particles and cancer cells samples were carried out to demonstrate the effectiveness of this method. Selectively manipulating a certain size of particles from a mixture solution was also achieved. Due to the very high pressure-driven flow switching, as many as 300 target cells per minute can be isolated from the sample solution and thus is particularly suitable for manipulating very rare target cells. The device is simple, automatic, and label-free and particularly suitable for isolating single cells off the chip one by one for downstream analysis.

## 1. Introduction

Recently, single cell analysis has attracted much interest in cell research fields [1,2]. Such a shift from cell analysis based on a population of cells is mainly due to the heterogeneous gene expression and behavior even in the same type of cells [3,4,5,6]. Single cell analysis, on the contrary, can clearly discover the biological behavior of one individual cell and is playing an increasingly important role in the fields of molecular biology, pathology, immunology, plant biology, stem cell biology, medical diagnostics, drug screening, and so on [7,8] and life sciences ranging from immune therapy and cancer research to tissue engineering and regenerative medicine [9,10,11,12,13]. 

For single cell analysis, isolating or trapping a single cell of interest from a cell population is the first important step. If more analysis, such as Polymerase Chain Reaction (PCR) or DNA sequencing, is further needed on the target cell, an ability to recover the cell for the trapped position is also demanded. For such purposes, therefore, single cell manipulation, defined as placing one cell in a desired place, is greatly needed. 

Several single cell manipulation methods have been proposed. Conventionally, single cell isolation is achieved by using a glass capillary tube (with an inner diameter slightly smaller than that of the cell) under a microscope. This method is simple but tedious and time-consuming. Additionally, care must be taken in order to avoiding damaging the cell’s integrity, which might be caused by the manual operation with a sharp glass tip. In response to an increasing demand for reliable, simple, and even automatic single cell operation, many efforts have been focused on single cell manipulation with a microfluidic chip. Generally, the methods can be categorized into label-based and label-free methods. The most commonly used label-based method is to use an external magnetic field to manipulate the magnetically labeled cells [14,15,16,17]. While this method has fewer side effects on the cells, it involves the use of a biomarker, which might not be available for the cells of interest. For some cells, the attached beads may interfere with the differentiation and expansion of the cells in vitro or in vivo [18].

For the various label-free approaches, they normally utilize different forces to, electrically [19,20,21,22], optically [23,24,25,26], hydraulically [27,28,29] or acoustically [30,31,32], manipulate the cells one by one. Trapping and positioning a single cell followed by various on-chip biochemical analyses have also been demonstrated recently [21]. More details about the above methods can be found in several review papers [33,34,35]. 

Dielectrophoresis (DEP) is a powerful method for both cell separation and single cell manipulation. Normally, AC-DEP is applied for cell trapping in order to avoid causing side effects to the cells. From the aspects of single cell manipulation throughput, the number of isolated single cells can be easily increased by increasing the number of traps. For this method, however, the biggest shortcoming is that it cannot freely move the cells off the chip. Therefore, the application of this method in recovering cells is greatly limited. Manipulating cells with optical or acoustic force is also an efficient method with the distinct advantage of precise control of individual cells. For the optical tweezers technology, it suffers from the disadvantages of low throughput and possible damages to the cells due to the applied concentrated laser beam [36]. Acoustic tweezers are a less invasive method for single cell manipulation [37] due to the low power density of the applied wave compared to optical tweezers. Similar to optical tweezers, it is powerful in trapping single cells in a desired position. The trapped cells, however, can hardly be recovered or isolated off the chip one by one for further analysis, such as PCR or DNA sequencing. Furthermore, the moving velocity of the cell under the acoustic force is relatively small [29], resulting in a low throughput on single cell isolation.

Cells can also be hydraulically trapped and isolated from each other. This method, known as hydrodynamic trapping, can automatically trap the cells in microstructures one by one by designing channels with different flow resistances. The working principle of this method is based on different fluidic resistances among the channels. A cell will be transported along the streamlines to the channel that has a smaller fluidic resistance firstly. Once the cell is blocked at the first trap, the fluidic resistance in this channel becomes larger than the other channels, and the next cell will move to the second channel. In this way, single cell isolation is achieved. For this method, the size of the trapped cells is determined by the size of the trap. Only particles with a size smaller than that of the trap can be trapped. Compared with the other methods, hydrodynamic trapping has the unique advantages of simple fabrication and application without using bulky instruments such as lasers, transducers, or signal generators [27]. The commercially available system C1™ (Fluidigm Corporation, South San Francisco, CA, USA) exemplify the power of this technology. Similar to the dielectrophoresis method, the throughput of this method strongly depends on the number of trapping channels [27]. For this method, however, neither selective trapping nor controllably releasing a specific cell is allowed.

To overcome the various shortcomings of the current single cell manipulation technologies, a novel microfluidic lab-on-chip device that can automatically and selectively manipulate target cells at single cell level was developed. The device is composed of a microfluidic chip, mini solenoid valves with negative-pressurized soft tubes, and a LabView^®^-based data acquisition device. Once a target cell is detected, a voltage signal is generated and then used to actuate the solenoid valves and open the negative-pressurized tubes placed at the ends of the collecting channels. Unlike other negative pressure-driven flows, the large pressure difference between the inlet and target wells is quickly balanced by the three-way solenoid valve. As a result, a switching flow with a high velocity yet short working time is achieved. Additionally, the collecting wells are blind-end, which enables transport of the waste cells directly to the outlet well without warring about the waste cells being transported to the collecting wells. This design greatly favors the easy operation of the device because there is no need to balance the liquid levels among the different wells. This method is simple, automatic, and particularly suitable for recovering or isolating single cells off the chip one by one for downstream analysis.

## 2. System Setup and Its Working Principle 

### 2.1. System Setup

**Figure 1:**
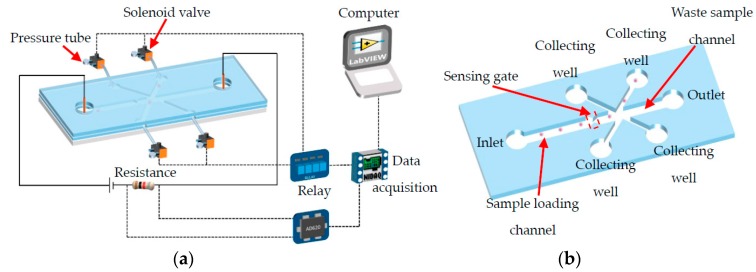
A schematic diagram of the system (**a**) and the structure of the microfluidic chip (**b**).

**Figure 2:**
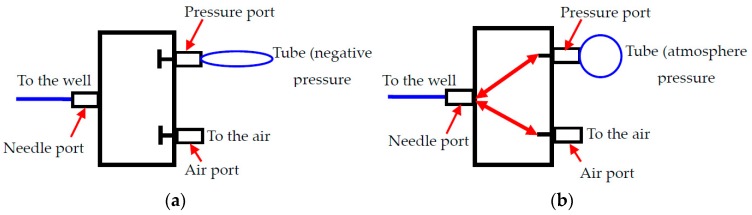
Two working modes of the valve, (**a**) before actuation and (**b**) after actuation.

As is shown in Figure 1a, the single cell manipulating system consists of a microfluidic chip, an electrical signal amplifier (AD620), four 2-position-3-way solenoid valves (HXL 170DC12V, FS Power Technology Co. Ltd., Fujian, China) with relays (SRD-12VDC-SL-C, HYNCDZ, Guangzhou, China), and a data acquisition device (NI USB6259, National Instruments Corporation, Budapest, BU, Hungary). The microfluidic chip can be divided into a sample loading channel, a sensing gate, a cell collecting channel, a waste sample channel, and corresponding end wells, as shown in Figure 1b. The inlet and outlet wells are open to the air. For the wells at the ends of the collecting channels, they are blind-end. There is a hollow needle inserted in the wells of the collecting channels with a tube connected to one port of the solenoid valve (known as the ‘needle’ port). The other two ports are open to the air (known as the ‘air’ port) and connected with the negative-pressurized soft tube (known as the ‘pressure’ port), respectively. There are two working conditions for the valve. In the normal close condition (before actuation), the ‘needle’ port, the ‘pressure’ port, and the ‘air’ port are separated from each other. Once it is powered on, the three ports are connected to each other. The details of the working mode of the valve are shown in Figure 2.

### 2.2. Operating Procedures and Working Principle

The idea of single cell manipulation can be simply summarized as detecting it and then collecting it. The detailed working process is as below.

Firstly, the inlet and outlet wells were added with the same amount of phosphate buffered saline (PBS, pH = 7.5). Afterwards, a certain amount of sample solutions were added into the inlet well and electrodes were inserted in the inlet and outlet wells. Direct current (DC) voltages of 12 V were applied. Finally, the valve assemblies were connected to the collecting wells. In this way, the system shown in Figure 1a is ready for use. Since the sample solution was only added into the inlet well, there is a liquid level difference between the inlet and outlet wells. As a result, the particles or cells in the sample solution will be driven to move by both pressure-driven flow and electroosmotic flow. Since the collecting well is blinded and the needle port is also closed when the valve is powered off, the sample solution will only flow from the inlet well to the outlet well. In this way, placing a valve in the collecting channel to control the flow is avoided.

When a particle passing the sensing gate (shown in Figure 1b), an electric resistance is generated due to the different resistivity of the particle and the electrolyte solution full of the channels [38,39,40,41]. The resistance change will generate a voltage pulse (known as the RPS signal) whose magnitude is proportional to the size of the cell. The detected signal is amplified by the amplifier and then input to the data acquisition device and the computer. A custom-made LabView^®^ program records the magnitude of the detected signal and compares it with the pre-calibrated reference value, which represents the size of the cell passing the sensing gate. When the magnitude of the detected signal is larger than the pre-calibrated value, it means that a target cell is detected. Then the computer will automatically send a signal to the data acquisition device, which will in turn power the solenoid valve on via the relay. Once the valve is activated, the three ports will be open and connected to each other. This will induce a pressure-driven flow in the collecting channel through the needle port. Such a pressure-driven flow, however, will only last for a very short period of time because the airport is opened at the same time. By using such a pressure control method, an instantaneous pressure-driven flow with high velocity can be achieved. This is beneficial in that switching more than one cell in one channel due to a long flow time is avoided. In other words, the cell density of the sample solution and thus the throughput can be improved in this way. After a certain amount of delay (adjustable), the solenoid valve will be powered off again, and the first cell is already manipulated to the collecting well. The other cells followed the same process. In this way, automatic single cell manipulation can be achieved. 

It should be noted that a reference value for the specific target particles (or cells) can be determined by measuring the RPS signals of the pure particle (or cells) solution in advance. Due to the uneven size distribution of the cells, the magnitude of the detected signals is also not the same value. Therefore, the reference value should be set as the smallest value of the measured signals.

**Figure 3:**
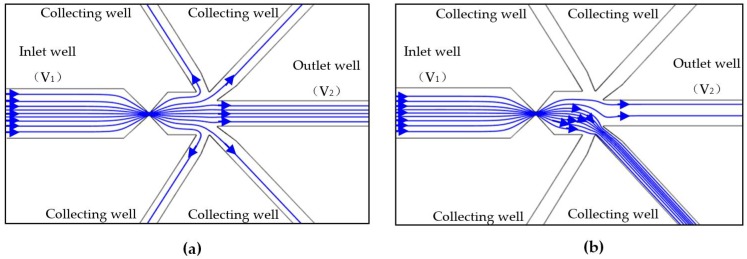
Solution flow before (**a**) and after (**b**) the actuation of the valve (*V*_1_ and *V*_2_ are the voltages applied in the wells and *V*_1_ = 12 V, *V*_2_ = 0 V. Zeta potential of the wall ζ_w_ = −50 mV, ε_r_ = 80).

Figure 3a,b show the simulation results of the solution flow before and after the actuation of the valve. Before valve actuation, all of the sample solution flows to the outlet well under the electric field and hydraulic pressure (Figure 3a). This is because the collecting channels are closed by the blind-end wells. When the valve is actuated, the sample solution is directed into the collecting channel, as shown in Figure 3b. After a pre-set time delay, the valve is powered off and the sample solution will continue to flow to the outlet well. Since there is a constant pressure difference between the inlet and the collecting wells, the collected cell will not escape from the collecting channel once isolated. Selective manipulating single cells can also be achieved if the cells in the sample solution have size differences. The thing is that cells with different sizes will generate signals with different magnitudes. By setting different reference values, cells can be selectively isolated into the collecting channels.

## 3. Experimental

### 3.1. Microfluidic Chip Design and Fabrication 

The microfluidic chip (Figure 1b) was designed with AutoCAD^®^ software firstly and then transformed to a chrome mask (Shenzhen Qingyi Photomask Co. Ltd., Shenzhen, China). The widths of the sample loading channel and waste sample channel are 200 μm and 100 μm, respectively. For the collecting channel, its width is 50 μm. In this study, two different sensing gates were designed. One is 15 μm wide and 8 μm long and is used for manipulating particles with diameters less than 15 μm. The other one is 25 μm wide and 10 μm long and is used for manipulating the cancer cells with an average diameter of about 20 μm. All of the channels are 25 μm in height. 

By using the soft lithography method [42] and a negative photo-resist (SU-8 2025, MicroChem Co., Newton, MA, USA), the master for rapid prototyping of the PDMS microstructure was fabricated on a silicon wafer substrate (4″ N/PHOS, Suzhou Yanshuo Company, Suzhou, China). In this study, blinded collecting wells should be fabricated in order to avoid the pressure-driven flow in the collecting channels when the solenoid valves were powered off. A novel fabrication method was developed to fabricate such wells. The procedures are as follows. Once the silicon master was obtained, iron cylinders with a diameter of 1.5 mm and a height of 4 mm were placed on the positions where the collecting wells are on the master. Then, a permanent magnet was placed under the silicon substrate. As a result, the ion cylinders can be tightly fixed on the silicon wafer. Afterwards, liquid polydimethylsiloxane (PDMS, Sylgard 184, Dow Corning, Midland, MI, USA) was poured over the master and cured in an oven for 2–3 h at 80 °C. The PDMS layer was then carefully peeled off from the master. In this way, blinded collecting wells with a height of 4 mm were fabricated. The inlet and outlet wells were punched with a hand puncher. Finally, the PDMS chip was bonded to a glass substrate (25.66 mm × 75.47 mm × 1.07 mm, CITOGLAS, Jiangsu, China) using a plasma cleaner (HARRICK PLASMA, Ithaca, NY, USA).

### 3.2. Sample Preparation

In this study, polystyrene particles and cancer cells (H1299) were used to demonstrate the effectiveness of this method. The polystyrene particle samples were prepared by mixing 5 μL polystyrene particles (Sigma-Aldrich, Shanghai, China) with 1 mL phosphate buffer solution (1 × PBS). The human lung NSCLC cell lines H1299 were obtained from the Cell Bank of Type Culture Collection of Chinese Academy of Sciences (Shanghai, China). Then, the human lung NSCLC cell lines H1299 cell were cultured in 1640 (Gibco, Waltham, MA, USA) at 37 °C in a humidified atmosphere of 5% CO_2_. These cell culture media were also supplemented with 10% fetal bovine serum (FBS, Hyclone, Logan, UT, USA), penicillin (100 U/mL), and streptomycin (100 μg/mL). To prepare the cancer cell sample for the experiments, a 10 μL cell culture suspension with the human lung NSCLC cell lines H1299 was mixed with a 1.5 mL phosphate buffer solution (1 × PBS).

### 3.3. Particle and Cell Motion Monitoring

During the experiments, the motion of particles and cells was monitored by an inverted optical microscope (Ti-E, Nikon, Tokyo, Japan) and recorded by a progressive charge-coupled device (CCD) camera (DS-Qi1Mc, Nikon). The camera was operated in video mode at a frame rate of 11.4 frames per second. The reading error in determining the cell position is about ±2 pixels, which corresponds to actual dimension of ±5.4 μm.

## 4. Results and Discussion

### 4.1. Automatic Single Cell Manipulation

**Figure 4:**
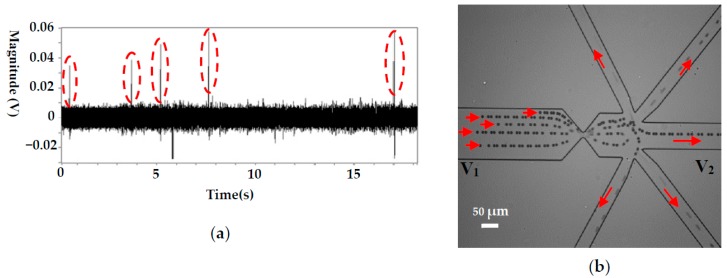
Single 10 μm particles manipulation for a pure particle solution, (**a**) the detected signals (sensing gate size: 15 μm × 8 μm), (**b**) the trajectories of 10 μm particles. (*V*_1_ and *V*_2_ are the voltages applied in the wells and *V*_1_ = 12 V, *V*_2_ = 0 V. *p* stands for the pressure at the collecting well when the valve is actuated and *p* = 100,093.96 Pa, the liquid level difference between the inlet and the collecting wells is about 1.25 mm).

**Figure 5:**
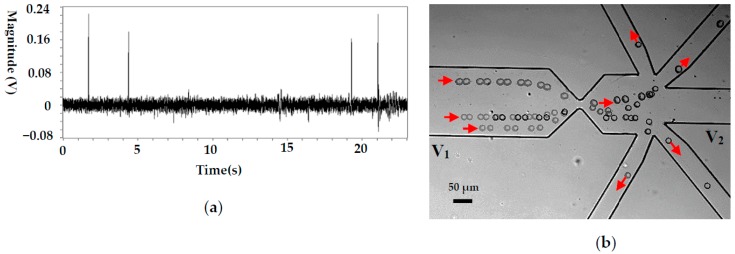
Single cancer cell manipulation for a pure cell solution, (**a**) the detected signals (sensing gate size: 25 μm × 10 μm), (**b**) the trajectories of cancer cells. (*V*_1_ and *V*_2_ are the voltages applied in the wells and *V*_1_ = 12 V, *V*_2_ = 0 V. *p* stands for the pressure at the collecting well when the valve is actuated and *p* = 100,093.96 Pa, the liquid level difference between the inlet and the collecting wells is about 1.25 mm).

To demonstrate the ability of this system on automatically manipulating single cells, pure particle and cell samples were employed, and typical results are shown in Figure 4 and Figure 5, respectively. Figure 4a shows the detected signals of 10 μm polystyrene particles. Each upward signal with a magnitude of larger than 0.03 V represents a particle passing the sensing gate. The downward peaks are caused by the LabView^®^ program, which is used to record and smooth the signals. It should be noted that the magnitude of the signals generated by the particles of the same size is not uniform, ranging from 0.035 to 0.06 V. This is caused by the different positions of the particles when passing the sensing gate where the electric field is not uniform [41]. The closer to the wall of the gate the particles are, the larger the magnitude of the signal. Since the noise level is less than 0.01 V, the reference value for 10 μm polystyrene particle manipulation can be reliably set as 0.03 V. In this way, each particle passing the gate will automatically trigger the actuation of the valve, which directs particles to the different collecting wells. The typical trajectories of polystyrene particles during this process are shown in Figure 4b. The trajectories were obtained by superposing a series of consecutive images of the moving particles. As can be seen from Figure 4b, the particle moves to the waste sample channel when the valves were not actuated. When the system puts into work, the particles will be directed to the different collecting channels one by one, and each channel has one particle. From Figure 4a, we can also find that the actuation of the valve does not increase the noise level or interrupt the signal detection process. Therefore, this system can be reliably used for single cell manipulation.

As a typical application, this system was applied to manipulate cancer cells (H1299) and the related results are shown in Figure 5. For the cancer cell, its size is relatively large (about 20 μm in diameter) and the magnitude of the detected signal is accordingly larger (between 0.18 and 0.22 V), as is shown in Figure 5a. Since the noise level is about 0.02 V, a signal-to-noise ratio (S/N) of 9:11 is achieved, which can guarantee reliable cell manipulation. Theoretically, the magnitude of an RPS signal is mainly determined by the volume ratio of the particle and the sensing gate. In this study, the volume of an H1299 cell is about 8 times that of a 10 μm polystyrene particle. This is the main reason for the larger magnitudes of RPS signals generated by H1299 passing a sensing gate with a larger volume (compared with the signals shown in Figure 4a).

Figure 5b shows the trajectories of the cancer cells in the channels. It can be seen that each cell is directed into separate collecting channels after passing the sensing gate. Some impurities in the solution continue to flow to the waste channel.

### 4.2. Selective Single Particle Manipulation

**Figure 6:**
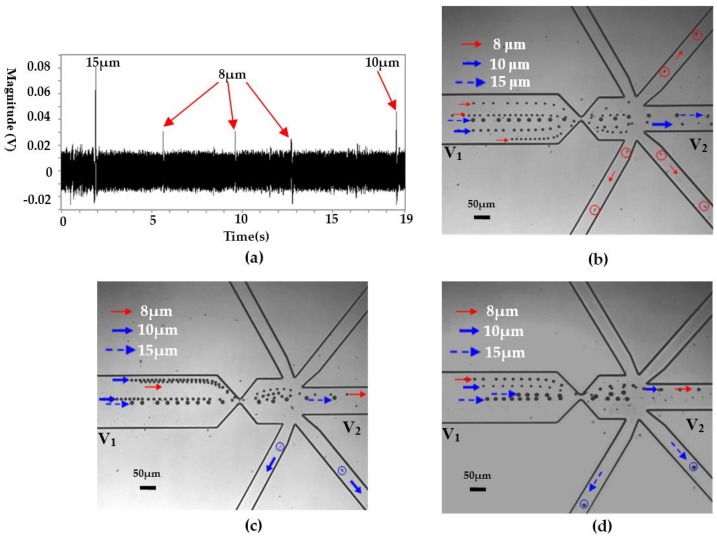
Selective manipulation of single particles based on size difference, (**a**) the detected signals (sensing gate size: 15 μm × 8 μm), (**b**) the trajectories of 8 μm particles, (**c**) the trajectories of 10 μm particles, (**d**) the trajectories of 15 μm particles. (*V*_1_ and *V*_2_ are the voltages applied in the wells and *V*_1_ = 12 V, *V*_2_ = 0 V. *p* stands for the pressure at the collecting well when the valve is actuated and *p* = 100,093.96 Pa, the liquid level difference between the inlet and the collecting wells is about 0.63 mm).

Theoretically, particles with different sizes will generate signals with different magnitudes. Furthermore, due to the high resolution on particle sizing of the RPS sensor [39], differentiation of particles with high resolution is possible. Therefore, selective manipulate single particles or cells is also possible. Figure 6 shows the typical results for selectively manipulating a certain size of particle from a mixed particle suspension. From Figure 6a, it is clear that there is an obvious magnitude difference for the three particles. For the 8 μm particle, its signal magnitude is about 0.03 V. For the 15 μm particles, its magnitude is about 0.08 V. As regards the 10 μm particles, the magnitude is in the range of 0.03–0.06 V, as is clearly shown in both Figure 4a and Figure 6a. Based on such magnitude difference and the preset value, the system can clearly identify the sizes of the detected particles and selectively isolate the target particle, as is clearly shown in Figure 6b–d.

It should be noted that the liquid level difference is smaller than that in single cancer cell manipulation test. As is explained above, the sample solution is driven by the combined pressure driven flow and electroosmotic flow. Due to the relatively low electric field applied across the channel, the electroosmotic flow is relative weak. The pressure driven flow is generated by the liquid level difference between the inlet and outlet wells. Since the cancer cell is larger in size (about 20 µm in diameter) than the particles, a relative high pressure-driven flow is needed in order avoid cell sedimentation.

### 4.3. Some Discussions on Flow Velocity and Throughput

In this study, negative pressure is used to control the flow switching and thus particles and cells movement between the channels. The switching velocity is mainly determined by the pressure difference (liquid level difference) between the inlet and the collecting wells. From this aspect, the negative pressure will also influence the throughput of this system. To know the switching velocity of the solution, the pressure difference should be determined after the valve is actuated.

Assuming that the deformed volume of the pressure tube is ∆*V*, the total volume of the pressure tube is *V*_1_, the total volume of the valve chamber and other spaces such as the needle and so on is *V*_2_, and the atmospheric pressure is *P*_0_. According to the ideal gas equation, the air pressure over the collecting well (*P*_1_) at the moment of opening the valve can be approximated as

(1) $$P_{1}\cdot (V_{1}+V_{2})=P_{0}\cdot (V_{2}+V_{1}-\Delta V)$$

The total pressure difference between the inlet and collecting well is the sum of (*P*_0_ − *P*_1_) and the hydrodynamic pressure difference caused by the different liquid levels of the inlet and collecting wells. For the flow velocity, it can approximately evaluated by doing numerical simulations with COMSOL (COMSOL Multiphysics 5.2a, COMSOL Inc., Shanghai, China). Table 1 and Table 2 shows the parameters used in the simulation and the results.

### 4.4. Efficiency and Throughput

Efficiency and throughput are two important parameters for evaluating the performance of the system. For the proposed system in this study, the two parameters are coupled with each other. If the sample is sufficiently diluted and the moving velocity of the cell is relatively low, the system will have enough time to isolate every cell into the collecting channels. That is, 100% efficiency can be achieved. When the cell density of the sample is high, the moving velocity of the cell has to be decreased in order not to isolate the cell next to the target cell together to the collecting channel. Otherwise, the density of the sample has to be decreased. The above two operations will both decrease the throughput. Here, we can approximately calculate the throughput per minute based on the parameters of the hardware and setting. 

For the measuring system described in this study, it will take 100 ms to activate the solenoid valve after receiving the detected signal. Due to the very high switching velocity, the working time for the valve is reduced as short as 100 ms. That is, 200 ms is required to complete one cell manipulation process. During this 200 ms detecting–sorting period, no other cells should come into the sensing gate. Otherwise, the undergoing switching process will isolate more than one cell into to one collecting channel. Therefore, the maximum throughput for the system presented in this paper is 300 cells per minute.

## 5. Conclusions

A microfluidic lab-on-chip device that can automatically and selectively manipulate target cells at single cell level is presented in this paper. A voltage signal generated when a cell passes the sensing gate is used to control the process. The switching velocity can be flexibly controlled by changing the deformation of the pressure tube and can reach as high as 1.58 mm/s, which allows for reliable manipulation. As a typical demonstration, automatically and selectively manipulate single particles and cells from either of pure or mixed sample solution were achieved. 

Due to the very high pressure-driven flow switching, as many as 300 target cells per minute can be isolated from the sample solution and thus is particularly suitable for manipulating very rare target cells, such as circulating tumor cells. The device described in this paper is simple, automatic, and label-free and particularly suitable for recovering or isolating single cells off the chip one by one for downstream analysis.

## Figures and Tables

**Table 1:** Parameters used in numerical simulations.

Parameters	Values	Units
Dielectric constant of the liquid ε_f_	80	-
Dielectric permittivity in vacuum ε_0_	8.85 × 10^−12^	F/m
Viscosity of the liquid μ	1 × 10^−3^	Pa.s
Density of the liquid ρ	1 × 10^3^	kg/m^3^
Zeta potential of channel walls ζ_w_	−50	mV
*V*_1_	0.25	cm^2^
*V*_2_	0.747	cm^2^
*P*_0_	1.01 × 10^5^	Pa
Liquid level difference △*H*	1.25	mm
Area of the well *S*	4	mm^2^

**Table 2:** Numerically approximated flow velocity under different deformed volume.

∆*V* (mm^3^)	Flow Velocity (mm/s)
0.14	1.580
0.12	1.379
0.1	1.176
0.08	0.974
0.06	0.773
